# Synthetic Approaches to Molecule‐2D Transition Metal Dichalcogenide Heterostructures

**DOI:** 10.1002/anie.202424932

**Published:** 2025-04-01

**Authors:** Sofiia Zuieva, Xin Chen

**Affiliations:** ^1^ Institute of Chemistry and Biochemistry Freie Universität Berlin Altensteinstraße 23a 14195 Berlin Germany

**Keywords:** Doping, Functionalization, Heterostructures, Janus TMDs, Transition metal dichalcogenides

## Abstract

The integration of 2D materials with molecular chemistry to create molecule‐2D material heterostructures presents a compelling strategy for advancing material design and applications. This approach provides precise control over the structure and properties of 2D materials, effectively addressing challenges in their production and fabrication. Among these, molecule‐2D transition metal dichalcogenide (mTMD) heterostructures have garnered significant attention due to their distinctive electronic, optical, and catalytic properties, as well as the intriguing emergent states and phenomena resulting from interactions with adjacent molecular and material layers. Achieving the desired electronic and optical properties in these heterostructures hinges on carefully controlling the interactions at the molecule/TMD interfaces. This minireview highlights recent progress in mTMD heterostructures, emphasizing the principles underlying interface interactions, molecular arrangement, and innovative synthetic methodologies.

## Graphical abstract

The integration of 2D materials with molecular chemistry enables precise control over material properties and enhances their functionality. Molecule‐transition metal dichalcogenide (mTMD) heterostructures are particularly promising due to their unique electronic, optical, and catalytic properties. This minireview highlights recent progress in mTMD heterostructures, focusing on interface interactions, molecular arrangements, and innovative synthetic methodologies.

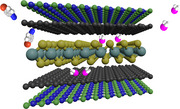

## 1 Introduction

Combining 2D materials with the immense versatility and precision of molecular chemistry to form molecule‐2D material (m2Dmat) heterostructures offers powerful means to tame and transform materials for innovation and applications across many fields. This concept is of great importance because it: a) offers efficient and controllable solutions to challenges in material production, fabrication, and application; b) enables atomic‐level control over the structures and properties of 2D materials; c) enhances understanding of the fundamental chemical properties and reactivity of 2D materials themselves; and d) enables new hybrid light‐matter quasiparticles (e.g., charge‐transfer excitons), which facilitate exploration of new physical phenomena and devices.^[^ ^1^, ^2^ ^]^ Since the discovery of graphene and other 2D materials, this strategy has enabled the creation of a wide range of m2Dmat heterostructures with diverse compositions and stacking configurations.^[^ ^3^, ^4^, ^5^ ^]^ In particular, heterostructures based on transition metal dichalcogenides (TMDs) have garnered significant attention due to their remarkable electronic, optical, and catalytic properties, along with their unique phase‐dependent characteristics.^[^ ^6^, ^7^, ^8^ ^]^ Additionally, these materials can exhibit fascinating emergent states and phenomena that arise from interactions with adjacent molecular and material layers. For example, the first evidence of hybrid charge‐transfer excitons was observed in WS_2_/tetracene heterostructures.^[^ ^2^ ^]^ These states and phenomena significantly influence the electronic structure, charge carrier dynamics, and spin dynamics, making these TMDs‐based heterostructures highly promising for applications in electronics, optoelectronics, catalysis, sensing, and beyond.^[^ ^9^, ^10^ ^]^

A diverse array of molecular components, such as organic dyes, electron donors/acceptors, photoswitches, spin‐crossover molecules, and polymers, has been used to fabricate molecule–2D TMD (mTMD) heterostructures.^[^ ^3^, ^11^ ^]^ These components can be integrated with TMD layers through chemical functionalization, self‐assembly, intercalation, or post‐synthesis transfer techniques.^[^ ^5^ ^]^ The fundamental forces that bind both components in the heterostructures are noncovalent or covalent interactions. The overall properties and functions of these multicomponent systems are determined by the intrinsic characteristics of both the 2D materials and molecular components, the interfacial interactions, and their spatial arrangement.

In the past 5–10 years, numerous reviews have addressed various topics in this field, including the functionalization of 2D materials,^[^ ^3^, ^12^, ^13^ ^]^ the fabrication and characterization of heterostructures,^[^ ^10^, ^14^, ^15^ ^]^ the physics of mTMD interfaces,^[^ ^6^, ^16^ ^]^ and potential applications.^[^ ^4^, ^5^, ^17^, ^18^ ^]^ However, there has been little emphasis on the impact of different stacking configurations and the density and spatial distribution of molecular components in many of these reviews. Additionally, there is an urgent need for systematic and in‐depth investigations into the synthetic chemistry of these assembly processes, which is essential for the rational design and precise regulation of heterostructures and their properties. This minireview will critically examine recent efforts in mTMD heterostructures, emphasizing the chemical interactions at the interfaces and the significance of spatial distribution and configurations of molecular components relative to the TMD layers. Emerging synthetic approaches for fabricating these heterostructures will be discussed, along with current insights into the factors influencing their electronic and optical properties. Additionally, fundamental challenges and future directions for the field will be outlined.

## 2 Fundamentals of mTMD Heterostructures

### 2.1 Structures and Properties of Pristine TMDs

Unlike graphene or black phosphorus (BP), 2D TMDs are binary compounds with the formula MX2, where M represents transition metals from group IVB (Ti, Zr, Hf), group VB (V, Nb, Ta), group VIB (Mo, W), group VIIB (Tc, Re), and group VIIIB (Pd, Pt), while X denotes chalcogens such as S, Se, and Te. Each TMD monolayer consists of a hexagonally packed metal plane sandwiched between two chalcogen planes, making the layer approximately 6–7 Å thick.^[^ ^19^ ^]^ Depending on the coordination of transition metal atoms, TMD monolayers can mainly crystallize in two different phases: the 2H phase (M in trigonal prismatic symmetry, D_3h_) and the 1T or 1T’ phase (M in octahedral or distorted octahedral symmetry, D_3d_). Under D_3h_ point group, the five degenerate *d* orbitals split into one singly degenerate state *a* ($d_{z^{2}}$), two doubly degenerated states *e’* (*d*_xy_, $d_{x^{2}}$ − 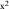), and *e* (*d*_xz_, *d*_yz_). The first two hybridize with each other with respect to the x‐y plane crossing transition metal plane, resulting in the fundamental bandgap in the *d*‐band and the energy reduction of the *a*‐subband. In comparison, under D_3d_ symmetry, *d* orbitals split into an e_g_ state ($d_{z^{2}}$, $d_{x^{2}}$ − 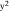), and a t_2g_ state (*d*_xz_, *d*_yz_, *d*_xy_). Each transition metal atom provides four electrons to fill the bonding states (σ); the nonbonding states are in the gap between the bonding (σ) and antibonding (σ*) bands. The remaining *d*‐electron count determines the preferred coordination type, which in turn dictates the material's properties. For example, in group VB (*d* ^1^) and group VIB (*d* ^2^), TMDs are commonly isolated in the 2H phase, whereas group IVB (*d* ^0^) and group VIIIB (*d* ^6^) TMDs are typically in the 1T phase. Group VII (*d* ^3^) adopts a distorted octahedral 1T’ phase. When the orbitals are fully occupied, as in 1T‐HfS_2_, 2H‐MoS_2_, and 1T‐PtS_2_, the materials exhibit semiconductor properties.^[^ ^20^ ^]^

For a given 2D TMD, one of the unique characteristics in 2D TMDs is the ability to undergo a phase transformation. For example, pristine MoS_2_ adopts the thermodynamically stable 2H phase with semiconducting properties. Upon intercalation or hot electron injection, it transitions to the octahedral 1T/1T’ phase to accommodate extra electrons, as evidenced by the appearance of 1T‐phase Raman modes (*J*_1_–*J*_3_), quenched photoluminescence (PL) intensity, and increased interlayer distance.^[^ ^21^, ^22^, ^23^ ^]^ However, the 1T/1T’ phase is metastable and can easily revert to the 2H phase through heating, prolonged storage, or electron depletion. In the past decades, a wide array of pure and mixed phase 2D TMDs have been successfully synthesized.^[^ ^24^, ^25^, ^26^ ^]^ The knowledge gained by phase engineering provides immense insights into fields such as energy storage, contact optimization, catalysis, and so on. This offers a theoretical foundation for tuning electronic properties via charge modulation methods such as doping, intercalation, and field effects.

While the electronic properties of 2D TMDs are primarily influenced by the transition metals, chalcogen atoms with higher atomic numbers have been found to reduce the bandgap with MoS_2_, MoSe_2_, WS_2_, and WSe_2_ are 1.76, 1.43, 1.95 eV, and 1.62 eV, respectively.^[^ ^27^, ^28^ ^]^ In the case of PtX_2_ monolayers, indirect bandgaps of 1.68, 1.18, and 0.40 eV for PtS_2_, PtSe_2_, and PtTe_2_, respectively.^[^ ^29^ ^]^ Due to quantum confinement in the out‐of‐plane direction, the bandgap varies significantly with the number of layers, with a transition from direct to indirect gap as the layer count increases from monolayer to multilayer. These varying bandgaps result in significant differences in the optical properties of 2D TMDs. For instance, semiconducting TMDs can absorb photons across the visible to near‐infrared spectrum.^[^ ^27^ ^]^ Additionally, reduced dielectric screening and 2D confinement enable the formation of stable excitons, even at room temperature.^[^ ^30^, ^31^ ^]^ Higher‐order excitonic quasiparticles such as trions, formed by the interaction between excitons and charge carriers, have also been observed in monolayer TMDs.^[^ ^30^ ^]^ Therefore, 2D TMDs have been appealing for excitonic physics and collective states. The population of trions in the PL process has been used as a read‐out to probe the charge transfer in many mTMD systems.^[^ ^32^, ^33^ ^]^ In addition to intrinsic electronic structures, lattice defects significantly influence the properties of 2D TMDs. Defects such as chalcogen vacancies and grain boundaries create trap states within the bandgap and serve as scattering centers, reducing charge carrier mobility and PL quantum yield.^[^ ^34^ ^]^ For example, MoS_2_ transistors without high‐*K* dielectric gate materials showed a low carrier mobility (1 cm^2^ V⁻¹ s⁻¹). However, the mobility can be significantly enhanced through defect and interface engineering.^[^ ^35^, ^36^ ^]^

On the other hand, vacancies and edges are significantly more reactive than the basal plane of a TMD layer, particularly as the size and thickness are reduced, making their effects more dominant. This increased reactivity arises because the basal plane consists of terminal chalcogen atoms without dangling bonds, while vacancies and edge sites feature unsaturated transition metal atoms capable of coordinating with ligands bearing lone electron pairs, such as thiols.^[^ ^37^, ^38^ ^]^ Numerous studies have shown that the edges and defects of 2D TMDs are critical factors in determining their catalytic reactivity. For instance, thin‐layered MoS_2_ exhibits significantly enhanced catalytic activity compared to its bulk counterpart, primarily due to its larger specific surface area and greater exposure of active sites. Additionally, 1T‐phase MoS_2_, produced via lithium intercalation and exfoliation, demonstrates substantially improved activity over 2H‐phase MoS_2_, likely due to enhanced electrode kinetics, superior conductivity, and a higher density of catalytically active sites.^[^ ^39^ ^]^

### 2.2 Principles of Molecule–TMD Interactions

Chemical principles for the assembly of mTMD hybrid structures generally involve integrating molecular components with TMD layers through either noncovalent or covalent interactions.

#### 2.2.1 Noncovalent Molecule–TMD Interactions

The atomically smooth surfaces and absence of dangling bonds in layered TMDs allow molecules to form high‐quality crystalline films with atomically sharp interfaces through self‐assembly, often without the need for covalent bonding. The self‐assembly of molecules is driven by intermolecular interactions (e.g., electrostatic interactions^[^ ^40^ ^]^ hydrogen bonding)^[^ ^41^ ^]^ and the relatively weak interactions between the molecules and the 2D material at the interface. Such noncovalent interactions at molecule‐TMD interfaces include van der Waals (vdW) forces,^[^ ^42^ ^]^ dipole–dipole interactions,^[^ ^43^ ^]^ and π–π stacking,^[^ ^44^ ^]^ offering significant versatility in designing mTMD heterostructures. Moreover, they eliminate the stringent requirements and limitations of lattice matching inherent in conventional epitaxy techniques.^[^ ^45^ ^]^ Many techniques have been applied to integrate noncovalent mTMD heterostructures, including physical vapor deposition (PVD),^[^ ^46^ ^]^ molecular beam deposition,^[^ ^40^ ^]^ spin‐coating,^[^ ^47^ ^]^ dip‐coating,^[^ ^48^, ^49^ ^]^ stamping,^[^ ^50^ ^]^ and so on. A recent study demonstrated a solvothermal method for the direct growth of highly crystalline, oriented 2D polymer films on a monolayer MoS₂ substrate, highlighting the role of the TMD layer as a template in the noncovalent polymer/TMD heterostructures.^[^ ^51^ ^]^ By combining these assembly methods with deterministic transfer techniques, various organic dye/photoswitch/electron donors and acceptors based on noncovalent mTMD heterostructures with disparate stacking orders have been fabricated. These will be discussed in detail in Section 3.

For these noncovalent mTMD heterostructures, electronic and optical properties are primarily governed by two interfacial processes: charge transfer and dipole–dipole interactions. Charge transfer occurs when the energy differences between the molecular dopant's lowest unoccupied molecular orbital (LUMO) and highest occupied molecular orbital (HOMO) and the conduction band minimum (CBM) and valence band maximum (VBM) of the 2D TMD are appropriately aligned: when the dopant's LUMO lies below the TMD's CBM, electrons can transfer from the TMD to the molecule, causing electron depletion and hole hopping (p‐type doping) in the TMD; conversely, when the dopant's HOMO lies above the TMD's VBM, electrons can transfer from the molecule to the TMD, resulting in electron accumulation (n‐type doping) in the TMD. Such energy level alignment determines both the direction of charge transport and the energy barriers for charge carrier tunneling across the interface. These energy barriers, in turn, govern the tunneling rates and interface resistance. Consequently, the energy level alignment plays a critical role in defining the electronic properties of mTMD heterostructures and related electronic device applications.

Over 50 molecular dopants, including benzyl viologen (BV, HOMO −11.9 eV)^[^ ^52^ ^]^ and tetracyanoquinodimethane (TCNQ, LUMO −5.3 eV),^[^ ^53^ ^]^ have been reported as effective n‐type and p‐type dopants. By tailoring dopant structures to modulate their HOMO/LUMO levels, the electronic properties of mTMD heterostructures can be precisely tuned. Additionally, dynamic photoinduced charge and energy transfer at molecule/TMD interfaces enriches the tunability of charge transfer scenarios by enabling external stimuli to further modulate these systems. While this process minimally affects charge carrier density in the dark, it can induce significant changes in optical properties, such as enhanced photoluminescence.

Dipole–dipole interactions arise at the interface when the static or transition dipole moments of aligned molecules on the TMD surface act as nanoscale electric gates, inducing local charge carrier redistribution. For a measurable macroscopic gating effect, the molecular components must be assembled in a long‐range ordered fashion, with their dipole moments aligned in the same direction. When the molecular dipoles are oriented perpendicular to the TMD surface, the resulting vertical electric fields can induce significant changes in the TMD's work function, detectable as variations in charge carrier concentration. The Zhao group provided a compelling example by fabricating hybrid van der Waals heterostructures (vdWHs) composed of photochromic azobenzene (AZO) molecules and 2D semiconductors such as MoS₂ or black phosphorus (BP). In the AZO/MoS_2_ hybrid system, light‐tunable Raman and PL spectra of monolayer MoS_2_ were observed, driven by switchable doping from AZO molecules. The light‐induced geometric transformation of AZO molecules redistributes their dipole moments on the 2D surface, effectively enabling light to remotely control the carrier concentration in the 2D semiconductor.^[^ ^54^ ^]^

In some cases, both charge transfer and dipole–dipole interactions coexist and collectively contribute to the modulation of the electronic properties of TMDs. Najmaei and coworkers demonstrated that molecular monolayers functionalized with various groups, including amine (─NH_2_), methyl (─CH_3_), fluoro (─CF_3_), and thiol (─SH), were used to modify substrates. Monolayer MoS_2_ was then transferred onto these functionalized substrates to fabricate transistor devices. Compared to devices using non‐functionalized substrates, the devices with functionalized substrates exhibited shifts in the transistor's threshold voltage and changes in the carrier density of the MoS_2_ layer. These effects were attributed to the built‐in dipole fields of the functional groups: positive dipoles (─CH_3_ and ─OH) depleted carriers in the MoS_2_ conduction band, while negative dipoles (─NH_2_, ─SH, and ─CF_3_) injected carriers into the MoS_2_ channel.^[^ ^55^ ^]^

Modulating the carrier density of these noncovalent heterostructures through interface charge transfer and dipole‐dipole interactions is also a direct and effective concept for tuning the optical properties of TMDs. This is because the optical response of 2D TMDs is fundamentally governed by the generation of excitons, subsequent charge transfer, and recombination processes, where carrier concentration and exciton lifetimes are playing critical roles. For instance, Mak and coworkers demonstrated that interfacing the p‐type dopant F_4_TCNQ with single‐layer MoS_2_ significantly enhances PL intensity, likely due to the suppression of trion formation.^[^ ^30^ ^]^

#### 2.2.2 Covalent Molecule–TMD Interactions

Covalent bonding involves strong, often irreversible interactions that can significantly alter the properties of the TMDs.

##### Defect engineering

**Figure 1:**
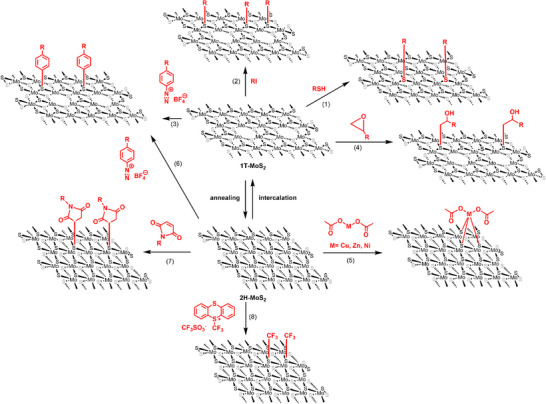
Synthetic strategies for covalent functionalization of 2D TMDs using MoS_2_ as a model system. For activated 1T‐phase MoS_2_: (1) defect engineering with organic thiols, (2) reactions with organohalides, (3) reactions with aryl diazonium salts, and (4) reactions with epoxides. For pristine 2H‐phase MoS_2_: (5) reactions with metal acetates, (6) reactions with aryl diazonium salts, (7) click chemistry with maleimides, and (8) reactions with electrophilic trifluoromethylation reagents.

Due to the inert nature of the TMD basal plane, covalent attachment of functional moieties is primarily achieved at chalcogen vacancy sites through a defect engineering approach. In this method, organic thiols coordinate with unsaturated transition metal sites, filling and functionalizing the vacancies (Figure 1, pathway 1). Subsequent annealing cleaves the C─S bonds, resulting in a vacancy‐fixed film.^[^ ^12^, ^56^ ^]^ Our group has shown an approach to controllably modify the sulfur vacancies (SVs) of MoS_2_ nanosheets using *para*‐substituted thiophenols. The degree of covalently modified SVs can be tuned by varying the electron‐withdrawing strength of *para*‐substituents in thiophenols.^[^ ^37^ ^]^ This approach, along with the obtained phenomenological structure–property relationships, provides a practical way to address the vacancies and improve the quality and properties of 2D TMDs. For the defect engineering of substrate‐supported TMD monolayers, the Zhao group utilized deep level transient spectroscopy (DLTS) to electrically characterize defect states in monolayer metal‐organic chemical vapor deposition (MOCVD)‐grown MoS_2_ on complementary metal‐oxide‐semiconductor (CMOS)‐compatible substrates.^[^ ^34^ ^]^ They identified a shallow trap state (T1) caused by hybridization of neighboring sulfur vacancy pairs, supported by STEM imaging and DFT calculations. Their study demonstrates DLTS as a sensitive, nondestructive technique for mapping defect energy states, paving the way for advanced defect engineering and improved performance in 2D semiconductor devices.

##### Reductive addition

Another widely applied covalent strategy involves the reductive activation of TMDs using *n*‐butyllithium or Na/K alloy, followed by the reaction with electrophiles such as alkyl halides,^[^ ^57^, ^58^ ^]^ diazonium salts,^[^ ^59^, ^60^, ^61^ ^]^ and epoxide^[^ ^62^ ^]^ (Figure 1, pathways 2–4). By leveraging different reaction reagents and sequences, Chen et. al demonstrated the bisfunctionalization of MoS_2_ nanosheets with alkyl chains and aryl groups.^[^ ^57^ ^]^ The negative charge density on activated MoS_2_ was found to be critical for covalent binding. Raman spectroscopy confirmed that functional group density could be precisely tuned. This strategy, applicable to both colloidal and substrate‐supported MoS_2_, lays a strong foundation for the development of multifunctional nanomaterials with customizable properties.

##### Direct addition

Direct covalent functionalization of pristine TMDs has been a challenging yet highly sought‐after topic. Recently, successful functionalization has been achieved using metal acetate salts,^[^ ^63^ ^]^ inorganic Lewis acid TiCl_4_,^[^ ^64^ ^]^ thiol‐maleimide “click” chemistry,^[^ ^65^ ^]^ and electrophilic trifluoromethylation reagents (Figure 1, pathways 5–8).^[^ ^66^ ^]^

Over the past decade, remarkable progress has been made in the functionalization of materials. These approaches enable precise structural modifications at specific binding sites and phases as needed. For instance, transport properties in as‐prepared 2D TMD layers are often hindered by charge traps, impurities, and short‐range defect scattering, with SVs being the most common defects. Thiol chemistry‐based defect engineering has been widely employed to repair these vacancies, enhancing charge carrier mobility. Additionally, eliminating defect‐mediated nonradiative recombination through thiol functionalization provides an effective approach to improving PL intensity and QY in MoS_2_. For instance, Amani et al. demonstrated that passivating MoS_2_ defects using bis(trifluoromethane)sulfonimide (TFSI) increased PL intensity by 190‐fold. A maximum QY of 95% was extracted from the pump‐power dependence of calibrated luminescence intensity at a low pump intensity (10^−^ **^2^** W cm^−^ **^2^**), accompanied by a much longer lifetime of approximately 10 ns, compared to several tenths of a nanosecond in pristine MoS_2_.^[^ ^67^ ^]^ To assess their effectiveness, the degree of functionalization serves as a useful metric. The reaction conditions and degrees of covalent functionalization are detailed in Table 1. Among the widely used methods, functionalization with diazonium salts stands out as the most effective for both 2H and 1T (1T’) phases, achieving a maximum functionalization degree exceeding 30%.

**Table 1:** Summary of reaction conditions and degrees of covalent functionalization.

Entry	Reagents	TMDs Form^a)^	Reaction Condition	Degree of Functionalization (∼per chalcogen atom)^b)^	Ref.
1	Thiols	CVD ME CE	IPA, r.t./50 °C 48 h	28%	[37, 68]
2	Organo‐halides	CE	H_2_O/IPA, r.t. 0.5–12 h	16%	[57, 58]
3	Diazonium salts	CVD ME CE	H_2_O, r.t. 0.5–12 h	32%	[59, 60]
4	Epoxides	CE	H_2_O, 60 °C 48 h	10%	[62]
5	Metal salts M^II^(OAc)_2_ M = Cu, Zn, Ni	LPE	IPA, r.t. 0.5 h	10%	[63]
6	Maleimides	LPE	CH_3_CN, Et_3_N, r.t, 16 h	33%	[65]
7	Trifluoromethylation reagents	LPE	CH_3_CN, r.t, 16 h	4%	[66]

^a)^ TMDs form: CVD = chemical vapor deposition grown; ME = mechanical exfoliated; CE = chemically exfoliated; LPE = liquid/co phase exfoliated.

^b)^ Maximum value from reported literatures.

Comparing noncovalent and covalent interactions reveals that each has its own advantages and shortcomings. For instance, noncovalent assembly offers simplicity and ease of fabrication but comes at the cost of weaker interaction strength, stability, and site specificity, as well as control over surface coverage and packing density. In comparison, covalent assembly provides robust interfacial binding, relatively well‐defined binding sites, and precise control, but typically requires greater synthetic effort.

## 3 Molecules Incorporation on TMD Surfaces

**Figure 2:**
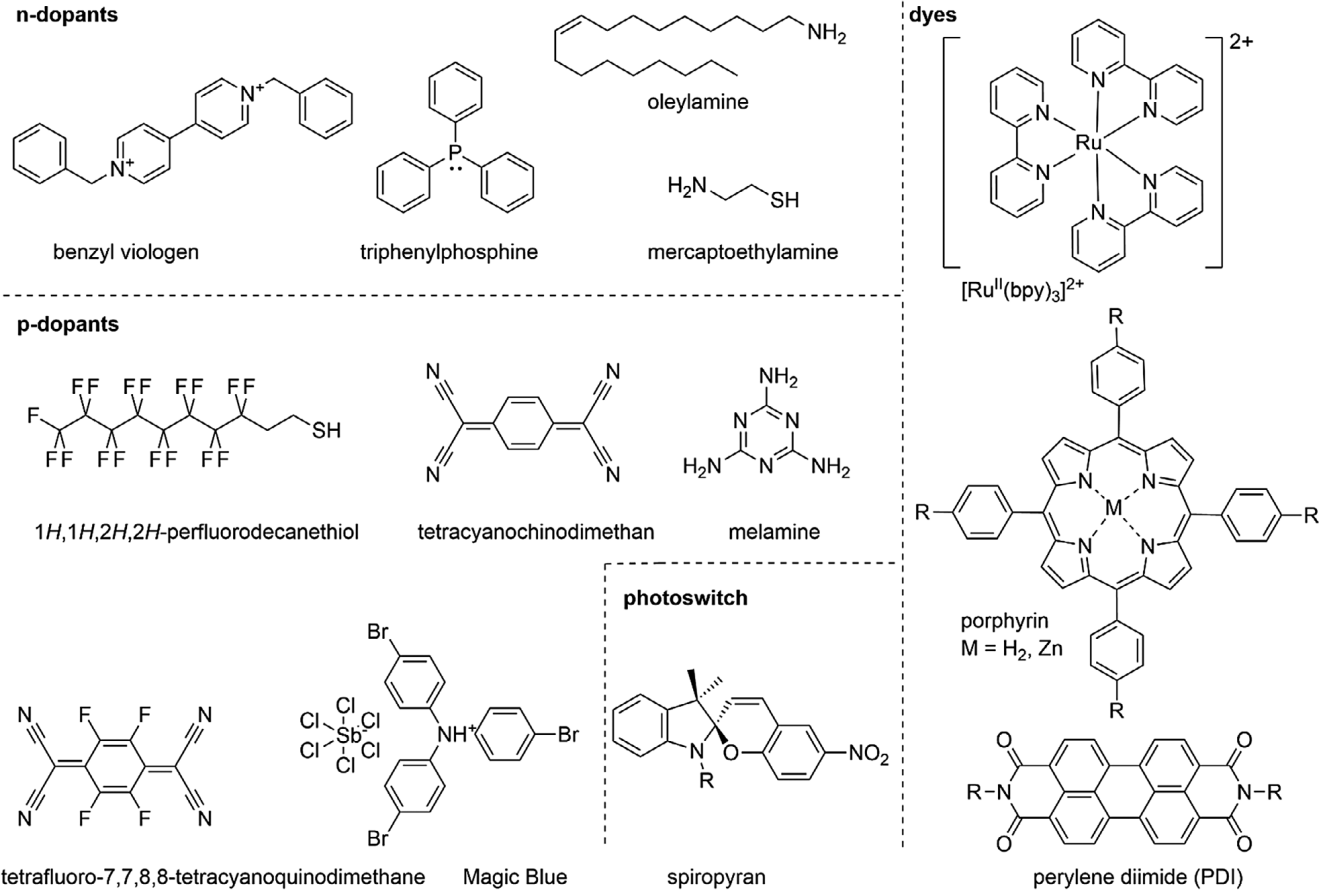
Representative functional molecular components used in the fabrication of mTMD heterostructures. The molecular components integrated into mTMDs serve as a functional layer, allowing for the tuning of the Fermi level, light absorption and emission, and surface properties (such as hydrophobicity and charge density) of 2D TMDs. They also enhance charge injection, improve electrical and mechanical properties, reduce contact resistance, and enable dynamic, remote control of various properties and functions.

Molecular components with complex functional units are often integrated onto TMD surfaces to achieve specific functions. The functional cores of molecules, such as organic dyes, electron donors/acceptors, photoswitches, and polymers (Figure 2), can be incorporated through noncovalent assembly strategies, or decorated with anchoring units for direct covalent immobilization, or interact with a surface‐pre‐tethered molecular buffer layer. This flexibility enables precise tuning of the desired properties and functionalities of the resulting hybrid systems. In particular, molecular dopants are chosen to ensure efficient charge transfer, while dyes are introduced to enhance light absorption or facilitate energy transfer. Photoswitches are incorporated for their ability to undergo reversible conformational and dipole changes, enabling dynamic control of the TMDs’ electronic and optical properties. For biological applications, biocompatibility factors such as toxicity, charge, and hydrophilicity are considered. Overall, molecular components are chosen based on their compatibility with TMD layers, taking into account factors such as energy level alignment, polarity, light absorption coefficients, and specific functionalities for the intended application. These factors ensure effective interaction with TMDs and optimized performance in electronic, optoelectronic, or biological contexts. These functional molecular components can be incorporated through noncovalent assembly strategies, or decorated with anchoring units for direct covalent immobilization, or interact with a surface‐pre‐tethered molecular buffer layer. The diverse types of molecular components and integration methods enable flexible tuning of the properties of mTMD heterostructures for tailor‐made functions.

### 3.1 Noncovalent mTMD Assembly

The direct interfacing of molecular components with TMD surfaces has been widely studied. Applications include tuning Fermi levels, optical properties, and surface characteristics, as well as enhancing charge injections, reducing contact resistance, and improving both electrical and mechanical properties. Furthermore, this approach enables dynamic remote control over various properties and functions. For example, the Hirsch group has recently demonstrated a noncovalently functionalized tungsten disulfide (WS_2_) with visible light‐absorbing, electron‐accepting perylene diimide (PDI) derivatives in the liquid phase.^[^ ^69^ ^]^ The significant fluorescence quenching observed in the WS_2_ PL of the PDI‐WS_2_ sample is attributed to an effective charge transfer across the WS_2_‐PDI interface. These interactions produce a long‐lived PDI^•−^‐WS_2_^•+^ charge‐separated state, which holds significant promise for photocatalytic applications. These topics have been thoroughly reviewed in existing literature.^[^ ^3^, ^9^, ^15^, ^70^ ^]^

After decades of searching for the most suitable molecular components for integrated functions, growing efforts have focused on precisely controlling the spatial arrangement and long‐range order of molecular films at 2D surfaces and interfaces, which is crucial for achieving the desired functions. The Bartlam group investigated the influence of the orientation of perylene derivatives on bilayer MoS_2_, exploring their role in organic–inorganic 2D heterostructures.^[^ ^71^ ^]^ Raman spectroscopy and low‐temperature optical spectroscopy revealed that the orientation of the perylene molecules on the MoS_2_ surface affects interlayer exciton formation, charge transfer, and PL properties, with perpendicular alignment enhancing electron accumulation in MoS_2_ and parallel alignment boosting organic–inorganic exciton populations. Their findings show that when perylene molecules are oriented perpendicular to the MoS_2_ surface, electron accumulation in MoS_2_ occurs, while a parallel orientation enhances the formation of organic–inorganic interlayer excitons. Additionally, power‐dependent PL measurements demonstrated how different orientations influence the saturation of interlayer states and the charge transfer efficiency, ultimately providing insights into optimizing these heterostructures for high‐performance optoelectronic and excitonic devices. This study underscores the importance of molecular orientation for controlling charge carrier dynamics and exciton behavior in 2D materials.

In the direction of controlling the orientation of molecular components, a novel strategy for doping monolayer MoS_2_ was developed using self‐assembled metal phthalocyanine molecules with orientation‐controlled axial ligands. This method enables precise control over the magnitude and orientation of molecular dipoles, allowing both p‐ and n‐type doping with charge carrier densities modulated up to 4.8 × 10^12^ cm^−2^. By focusing on the alignment of molecular dipoles rather than charge transfer, this approach offers a straightforward and highly adaptable solution to enhance the functionality of 2D materials for optoelectronic applications.^[^ ^72^ ^]^ Monolayer 2D polymers (2DPs), one‐molecule‐thick freestanding films of periodically linked monomers, exhibit tunable molecular‐level properties and can form heterostructures through van der Waals assembly, similar to 2D atomic crystals at a sharp pentane/water interface. Zhong et al. synthesized wafer‐scale 2D porphyrin polymer films with monolayer thickness.^[^ ^73^ ^]^ These films included metal‐organic frameworks with Cu^2^⁺ linkers and covalent organic frameworks with terephthalaldehyde linkers, with lattice structures and optical properties precisely controlled by monomer selection and polymerization chemistry. The resulting 2D polymers were employed to fabricate hybrid superlattices with molybdenum disulfide, showcasing their potential for applications in electrical capacitors.

One emerging class of mTMD heterostructures is called Janus‐type heterostructures, featuring top and bottom sides of material layers modified with different molecular components, named after the Roman god Janus with two faces looking in opposite directions. This out‐of‐plane asymmetry generates a built‐in vertical electric field due to the electronegativity difference between the top and bottom chalcogen atoms, inducing many new phenomena such as Rashba spin‐orbit coupling (SOC), vertical piezoelectricity, and weakened exciton binding.^[^ ^74^ ^]^ First‐principles studies of Janus WSSe reveal a Rashba parameter of up to 158 meV, attributed to its broken out‐of‐plane mirror symmetry, unlike pristine TMD monolayers where Rashba SOC is absent. This spin splitting shows significant potential for spin field‐effect transistors and valleytronic devices.^[^ ^75^ ^]^ In addition, Najmaei et al. reported out‐of‐plane piezoelectric responses in Janus TMD structures with dramatically enhanced vertical piezoelectric coefficients, making them ideal for energy harvesting, actuators, and nanoscale sensors, and expanding their applications in energy and mechanical systems.^[^ ^76^ ^]^ In the Janus TMD monolayers, the intrinsic vertical electric field induces strong Coulomb screening and reduces the overlap of electron–hole wave functions, suppressing the recombination process and thereby extending the exciton lifetime. This is particularly advantageous for optoelectronic and catalytic applications. Time‐dependent DFT calculations on Janus MoSTe predict exciton lifetimes of up to 1.31 ns, significantly longer than in conventional TMDs, enhancing their performance in photodetectors and light‐emitting diodes.^[^ ^77^ ^]^ A computational screening of a series of Janus TMDs reveals that WSSe monolayers can catalyze HER without external strain, with enhanced performance likely attributed to the presence of chalcogen vacancies and intrinsic lattice strain and electric fields from Janus asymmetry, which alter the crystal field and band structures.^[^ ^78^ ^]^ The intrinsic dipole in Janus TMDs, which can influence gas adsorption by either enhancing or weakening it depending on the polarization direction, makes them uniquely suited for sensor applications.^[^ ^79^ ^]^

A quaternary‐responsive WSe_2_‐based field‐effect transistors (FETs) were developed by Qiu et.al,^[^ ^80^ ^]^ integrating light‐responsive spiropyran (SP) molecules on the bottom and a ferroelectric co‐polymer layer (P(VDF‐TrFE)) on the top. These components enable independent and synergistic control of the output current through heat, light, and electric field, modulating charge transport and switching device polarity from n‐type to p‐type with 99% efficiency. This versatile approach offers a general strategy for engineering multifunctional FETs, advancing More‐than‐Moore technologies. In 2022, Weintrub's group overcome conventional dielectric limitations by suspending a 2D material (2DM) between two ionic liquids (ILs) with independently controlled potentials, creating an intense electric field exceeding 4 V/nm.^[^ ^81^ ^]^ This double‐sided ionic gating forms oppositely charged electric double layers (EDLs) on either side of the 2DM, enabling phenomena inaccessible to standard FETs. The high field strength, confirmed through electrical transport measurements, drives a semiconductor‐to‐metal transition in bilayer WSe_2_ and surpasses fields achievable in dielectric‐gated devices by an order of magnitude. This innovative approach opens new avenues for exploring extreme electric field effects in 2DMs.

### 3.2 Covalent mTMD Assembly

The aforementioned covalent functionalization strategies can be used independently or combined with other covalent or noncovalent approaches sequentially to introduce the target functional units. The Canton‐Vitoria's group demonstrated edge modification of exfoliated MoS_2_ with a 1,2‐dithiolane derivative bearing a porphyrin (H_2_P). The covalent linkage did not significantly alter the porphyrin's absorption or fluorescence. Transient absorption spectroscopy revealed a unique ping‐pong energy transfer mechanism between the porphyrin and MoS_2_. This study highlights the potential of transition‐metal dichalcogenides in photosensitization applications.^[^ ^82^ ^]^

In addition to direct immobilization, the functional unit can also be introduced through covalent or noncovalent interactions with surface‐tethered guiding molecules. For example, spin‐crossover nanoparticles (SCO‐NPs) were covalently grafted onto functionalized MoS_2_ layers to form a hybrid heterostructure. The process involved chemically functionalizing exfoliated MoS_2_ with 3‐iodopropyl(trimethoxysilane) (IPTS), followed by anchoring the SCO‐NPs through covalent bonding. This hybrid structure enhances luminescence, resilience, and conductance compared to pure SCO‐NPs. The SCO‐NPs’ spin transition, triggered by light or temperature, induces strain and a reversible change in the electrical and optical properties of the MoS_2_ layer.^[^ ^83^ ^]^ Another example details a Hamilton‐type recognition motif on the surface of 1T‐MoS_2_, enabling host‐guest interactions via hydrogen bonding for electrochemical sensing of barbiturates.^[^ ^84^ ^]^ The MoS_2_ is first functionalized with the Hamilton receptor, which then forms a host‐guest supramolecular pair with ferrocene‐labeled barbituric acid on the surface through hydrogen bonding interactions.

The surface‐tethered molecular components can also act as ligands, coordinating with the transition metal core to form inorganic complex/TMD heterostructures. By applying this concept, Chen et al. demonstrated an efficient catalyst for light‐driven hydrogen evolution reactions (HER) by covalently tethering ruthenium complex‐based photosensitizers (PS) to the basal plane of MoS_2_. Compared to the non‐tethered mixture, the photocathode with covalently linked components exhibited improved photocurrent generation, faster light on/off switching, and stable current density over 24 h. This enhancement is attributed to the covalent linkages, which reduce electron‐hole recombination and improve interfacial charge transfer, leading to better photocurrent performance.^[^ ^85^, ^86^ ^]^ In a similar fashion, cobaloxime‐anchored MoS_2_ nanosheets (MoS_2_–Co(dmgBF_2_)_2_) were developed via the coordination of cobalt on 4‐cyanobenzyl‐functionalized MoS_2_ nanosheet templates as electrocatalysts for the HER in acidic media. This enhanced HER activity is attributed to abundant active sites on both the edges and modified basal planes of the MoS_2_ nanosheets.^[^ ^87^ ^]^

Aiming for a defined allocation of molecular components, Chen et al. recently developed a covalent patterning method that combines bottom‐up surface chemistry with top–down lithographic techniques. This approach utilizes electron beam lithography (EBL) and diazonium chemistry for the covalent functionalization of CVD‐grown MoS_2_ monolayers. The resulting patterned MoS_2_ ribbons, with a minimum feature size of 2 µm, exhibit switchable properties, as confirmed by scanning Raman spectroscopy and PL mapping, offering great potential for advanced electronic and optoelectronic devices.^[^ ^61^ ^]^

## 4 Molecules Incorporation at 2D Interfaces

Encapsulating foreign components such as ions, atoms, and molecules within homo‐ or heterointerfaces might initially seem counterintuitive, as the molecular layer is concealed beneath the material layer, potentially limiting its interaction with external stimuli. However, the intercalation of 2D material layers and heterostructures has proven to be an effective strategy for tuning chemical, optical, electrical, and magnetic properties. Significant advancements have been achieved in this field, which have been thoroughly reviewed in recent studies.^[^ ^40^ ^]^ Leveraging the facile transferability of 2D TMD films, molecular components can be encapsulated using a top‐down, layer‐by‐layer approach.

### 4.1 Noncovalent Interface Engineering

Ion intercalation in layered materials typically induces phase transformations and slight expansion of interlayer spacing, enhancing their electrical and electrocatalytic properties. Recent advances have shown that intercalation with molecular surfactant ions can significantly enlarge the interlayer distance, forming organic/2D layered material (2DLM) superlattices with novel properties. Building on this, He and coworkers demonstrated a versatile electrochemical intercalation strategy to create 2D atomic crystal/functional organic molecule heterostructures. This approach integrates transition metal dichalcogenides (TMDs) like MoS_2_, WS_2_, and WSe_2_, known for exceptional charge transport, with organic semiconductors such as PTCDA, pentacene, and fullerene, which exhibit superior electronic and photonic characteristics.^[^ ^88^ ^]^ Cross‐sectional scanning transmission electron microscopy (STEM) with high‐angle annular dark field (HAADF) imaging confirmed the alternating layers of MoS_2_ and PTCDA, while layer‐by‐layer electron energy loss spectroscopy (EELS) provided evidence of strong interfacial electronic coupling. This method has been successfully applied to various 2DLMs and organic semiconductors, establishing a platform for fabricating heterostructures with diverse functionalities.

Controlling the height of van der Waals (vdW) gaps between 2D materials is inherently challenging due to the strength of vdW interactions. Seminal work by Liu and co‐workers presented a versatile approach to control van der Waals (vdW) gaps by pre‐adsorbing water molecules on material surfaces.^[^ ^89^ ^]^ By adjusting water vapor pressure, the vdW gap height in MoS_2_ homojunctions can be precisely tuned from 5.5 to 53.6 Å. This method extends to various 2D and 3D systems, allowing for tailored interlayer coupling, conductivity, and topology. Demonstrating its potential, a MoS_2_/gap/MoS_2_ diode highlights the practical utility of vdW gap engineering for modulating device performance across diverse vdW interfaces. DFT calculations show that water molecules spontaneously polarize on the MoS_2_ surface, creating a structured polarization in subsequent molecules. The resulting vertical polariton‐potential‐difference (PPD) enables the MoS_2_/gap/MoS_2_ homo‐gap‐junction to exhibit gap‐dependent diode characteristics with an ideality factor of 1, an ultrahigh rectification ratio (>10^5^), and excellent stability. Around the same time, our group developed a simple method to adjust the interface distance and properties of graphene/MoS_2_ (G/MoS_2_) heterostructures by varying functional groups on the graphene layer.^[^ ^33^ ^]^ By adjusting the backbone size of these functional groups, we can systematically tune the interlayer distance at the nanometer scale. An increased interlayer distance weakens the binding energy between the heterolayers, significantly lowering charge transfer efficiency and reducing the population of corresponding charge carriers in MoS_2_. The modulation of charge transport, in turn, significantly influences the PL emission of MoS_2_. This approach offers a versatile strategy to modulate interlayer spacing and charge transport, showcasing the potential of interface chemistry in optimizing vdW heterostructures.

### 4.2 Cross‐Linked Interface Engineering

The need for high structural stability and tunable properties at heterointerfaces to enhance performance, along with the limitations of vdW heterointerfaces, has led to the emergence of a new class of mTMD heterostructures that are covalently bonded. Adjacent material layers can be connected through bifunctional linkers or cross‐linked using surface‐tethered functional groups. Ippolito's groups demonstrated the use of dithiols to repair SVs in liquid‐phase exfoliated MoS_2_ flakes and covalently bridge adjacent flakes, enhancing charge transport.^[^ ^38^ ^]^ This approach achieved a reproducible increase by one order of magnitude in field‐effect mobility (*µ*_FE_), current ratio (*I*_ON_/*I*_OFF_), and switching time (*τ*_S_) for liquid‐gated transistors, reaching values of 10^−2^ cm^2^ V^−1^ s^−1^, 10^4^, and 18 ms, respectively. The following on multiscale analysis of charge transport in covalent MoS_2_ networks shows that hopping is the dominant mechanism. Aliphatic linkers lead to 3D variable range hopping, while aromatic linkers result in nearest‐neighbor hopping. Percolation theory explains the enhanced performance of devices with π‐conjugated molecules, due to improved electronic connectivity and additional percolation paths.^[^ ^90^ ^]^ These insights provide guidelines for optimizing charge transport in MoS_2_‐based devices.

Methods like layer‐by‐layer assembly, chemical vapor deposition, defect engineering, click chemistry, polymer cross‐linking, and plasma‐assisted functionalization offer versatile strategies for creating stable, covalently bonded heterostructures. Most examples have been reviewed in previous literatures.^[^ ^4^, ^13^, ^91^, ^92^ ^]^ The recent endeavor by Vázquez Sulleiro et al. demonstrated a covalent grafting method to attach MoS_2_ flakes to graphene in field‐effect transistors using a bifunctional molecule with maleimide and diazonium groups.^[^ ^93^ ^]^ This approach allows simultaneous functionalization of multiple devices, with the electronic properties dominated by the MoS_2_–graphene interface. The resulting p‐doped devices maintain charge mobility, and doping can be controlled. This method offers control over interlayer distance and chemical properties, enabling new functionalities and the production of many devices at once.

Notably, the homogeneity of their heterostructures is compromised by the random accumulation of MoS_2_ flakes on the graphene surface, posing challenges for further patterning and integration into high‐performance devices. In a recent study, we, however, showcased a novel bottom‐up approach for fabricating and patterning high‐quality G/MoS_2_ heterostructures.^[^ ^94^ ^]^ This method involves the use of heterobifunctional linkers with a diazonium end and a photolabile halogen end. The incorporation of photolabile linkers facilitates remote and efficient initiation and control of covalent interface assembly. This interface coupling, combined with the use of a pre‐patterned bottom layer, enables the precise transduction of both chemical information and topographic patterns from the bottom to the top layer of the heterostructures. The built‐up strain on graphene caused by the interface coupling is likely relieved through the antaratopic additions of H or OH groups to the top side of graphene,^[^ ^95^ ^]^ yielding a Janus graphene top layer. Our approach establishes a new paradigm for the design, fabrication, and integration of mTMD heterostructures and Janus‐type 2D layer.

## 5 Summary and Outlook

From the early advancements in organic semiconductors to the advent of 2D semiconductors and, more recently, synthetic 2D polymeric networks, it has become evident that no individual material class can fully address all technological demands. In particular, all‐2D vdW heterostructure devices suffer from challenges such as lattice mismatching problems and performance limitations. In this regard, combining molecular building blocks and chemistry with 2D materials to create heterojunctions has shown great promise in overcoming these limitations and propelling innovations beyond the combined properties of individual building blocks. In the last decades, extensive efforts have been devoted to fabricating molecular‐transition metal dichalcogenide (mTMD) heterostructures, exploring different material and molecular classes, stacking orders, and interface interactions. Despite this progress, many fundamental questions and practical challenges remain unresolved.

One major area of focus has been the noncovalent incorporation of molecular components onto the surface of TMD layers. Considerable advancements have been achieved in the fabrication of noncovalent mTMD heterostructures and the development of related proof‐of‐concept devices. However, achieving a long‐range order of molecular films at 2D surfaces continues to be a significant challenge. Furthermore, systematic studies are still needed to understand how spatial arrangement, including orientation of molecular components, interlayer distance, intramolecular proximity, and lattice disorder, impacts the properties and performance of these heterostructures. In terms of the fabrication process, the reverse approach, transferring 2D materials onto organic substrates, has seen limited exploration. The primary obstacle lies in finding an orthogonal solvent capable of efficiently removing poly(methyl methacrylate) (PMMA), a commonly used support layer during wet transfer, without compromising the integrity of the underlying organic layer.

For covalent anchoring of molecular components to the TMD surface, although significant efforts have been made to explore fundamental concepts, there is limited characterization evidence to confirm the presence of covalent bonding, particularly for those fabricated directly on the surface. The scalability of covalent approaches depends on both the scalability of TMDs and the efficiency of functionalization. Almost all reported covalent methods have been demonstrated using liquid‐phase or chemically exfoliated TMD nanosheets dispersed in solution. Advances in the solution‐phase production of 2D TMD nanosheets, along with improved size‐selection methods, have made it feasible to scale up the production of functionalized TMDs. However, challenges related to the homogeneity, dispersibility, and compatibility of these functionalized dispersions with subsequent device fabrication processes must be addressed for practical applications. Additionally, depositing functionalized materials onto substrates to ensure optimal interlayer and interface contact remains a significant challenge. Efforts combining chemical linkages with deposition techniques commonly used in the semiconductor industry may offer viable solutions. Improving the efficiency of covalent functionalization is also critical. This has been explored by enhancing the reactivity of TMDs (e.g., activating them with strong reductants) and by modifying the reactivity of molecular components through electron‐withdrawing or electron‐donating substituents. A better understanding of the reaction mechanisms and their dependence on TMD phases is needed to develop new chemical processing strategies that promote efficient binding. Another key consideration is the purity of functionalized TMD surfaces. While radical chemistry‐based approaches like diazonium salts and epoxide functionalization are efficient, they may produce polymerization‐induced side products. Thus, strict control over the functionalization process and precise characterization of the products are essential before application.

In addition, despite the high desirability of Janus molTMD heterostructures for their unique properties, research in this area is still in its early stages, with only a few experimental studies reported so far. In particular, Janus mTMD heterostructures realized by asymmetric covalent functionalization have yet to be reported. Moreover, experimental data on the feasibility and compatibility of covalent mTMD heterostructures in device applications is scarce, as is a comparison to noncovalent alternatives. Fundamentally, the degree of functionalization, and thus the surface coverage, remains low with covalent approaches. Key challenges include enhancing the reactivity of TMDs, understanding the topicity and regional selectivity of covalent binding, and improving surface coverage to achieve a closely packed, long‐range ordered molecular layer. Additionally, there are inherent trade‐offs between maximizing surface coverage and preserving the lattice integrity and properties, requiring careful consideration. The optimized performance of functionalized TMDs can be achieved by carefully tuning the degree of functionalization. Finally, to facilitate the integration of these materials into miniaturized devices, there is an urgent need for the development of efficient methods for high‐resolution covalent patterning of TMDs.

The encapsulation of molecular components has been studied for nearly two centuries due to its significant technological importance, particularly for energy storage in batteries and the production of highly conductive, lightweight carbon compounds. Despite these advances, the fabrication of controlled molecular 2D material heterostructures through intercalation remains challenging, primarily due to steric hindrance caused by the small distance between 2D layers. However, building on recent developments in nanofabrication and 2D material production, new methods for intercalating molecular components may soon become feasible. Exploring new bonding principles beyond vdW forces to revolutionize stacking paradigms offers a promising, yet largely unexplored solution. Due to the versatility of their molecular linkers, these mTMD heterostructures present a promising new direction for developing advanced heterostructures and creating confined nanospace.

Standing at the forefront of a new era in low‐dimensional chemistry, we are witnessing how molecular chemistry can shape the structures and properties of 2D materials. Furthermore, these intricate structures and the emerging physical phenomena can be harnessed to transform chemical reactions. A recent study by In Yoon et al. demonstrated that reactants confined between atomically thin sheets of graphene or hexagonal boron nitride experience pressures as high as seven gigapascals. This extreme pressure enables solvent‐free organic reactions that would not typically occur under standard conditions. Specifically, the study revealed that cyclodehydrogenation of hexaphenylbenzene, without the need for catalysts, and the oxidative polymerization of dopamine into sheet‐like crystalline structures were both facilitated by the high pressure within the graphene layers.^[^ ^96^ ^]^ These findings highlight the potential of 2D interfaces to drive novel chemical reactions.

## Conflict of Interests

The authors declare no conflict of interest.

## Data Availability

The data that support the findings of this study are available from the corresponding author upon reasonable request.
